# Real-World Safety and Efficacy of 156 U – 195 U OnabotulinumtoxinA in Adults With Chronic Migraine: Results From the REPOSE Study

**DOI:** 10.1186/s12883-025-04087-7

**Published:** 2025-05-06

**Authors:** Fayyaz Ahmed, Charly Gaul, Katja Kollewe, Ritu C. Singh, Katherine Sommer

**Affiliations:** 1 Spire Hesslewood Clinic, Hull York Medical School, Brough, Hull UK; 2 Headache Center Franfurt, Franfurt, Germany; 3 Medical School Hannover, Hannover, Germany; 4 AbbVie, Madison, NJ USA; 5 Marlow, SL7 1YA United Kingdom

**Keywords:** Chronic migraine, Efficacy, OnabotulinumtoxinA, Patient-reported outcomes, Safety, Real-world study, Headache

## Abstract

**Background:**

The phase 3 PREEMPT clinical trials confirmed the efficacy and safety of 155 U – 195 U onabotulinumtoxinA for individuals with chronic migraine (CM) and is the licensed dose in Canada and Europe. This analysis aimed to analyze the efficacy and safety parameters of 155 U – 195 U onabotulinumtoxinA in participants with CM from the real-world REPOSE study.

**Methods:**

REPOSE (NCT01686581) was a 2-year, prospective, observational, noninterventional, open-label study that described the real-world use of onabotulinumtoxinA in adults with CM in Europe. Participants received onabotulinumtoxinA approximately every 12 weeks and were monitored for 24 months after starting treatment. Data on participant-estimated mean headache-day frequency in the last month (MHD), Migraine-Specific Quality of Life Questionnaire (MSQ) scores, and adverse events (AEs) were collected at each treatment visit. Participants in the safety analysis population (those who received at least one dose of onabotulinumtoxinA) were stratified into two groups based on the dosage received at four or more treatment visits: 155 U onabotulinumtoxinA and 156 U – 195 U onabotulinumtoxinA groups.

**Results:**

A total of 641 participants were enrolled at 77 centers. Of those, 218 participants received 155 U ≥ 4 treatment visits, and 77 participants received 156 U–195 U onabotulinumtoxinA ≥ 4 treatment visits. Between-group baseline characteristics were similar. Reductions from baseline in MHD frequency were observed at both doses (156 U – 195 U range, -8.7 to -14.2 MHDs; 155 U range, -8.2 to -11.9 MHDs). Mean change from baseline in MSQ domain scores improved across administration visits for both 155 U onabotulinumtoxinA and 156 U – 195 U onabotulinumtoxinA groups. Treatment with 156 U – 195 U onabotulinumtoxinA was safe and generally well-tolerated with no new safety signals identified. Adverse drug reactions (ADR) were reported in 51/218 in the 155 U group and 10/77 participants in the 156 U – 195 U group; serious adverse drug reactions were 3/218 and 1/77, respectively. The most frequently reported ADR across both dose groups was eyelid ptosis, followed by neck pain, musculoskeletal stiffness.

**Conclusions:**

These real-world findings of the safety and efficacy of the 155 U – 195 U onabotulinumtoxinA doses are consistent with data from the PREEMPT clinical trials as a treatment option for CM patients.

**Trial registration:**

NCT01686581. Name of registry: ClinicalTrials.gov. URL of registry: Date of retrospective registration: September 18, 2012. Date of enrolment of first patient: July 23, 2012.

**Supplementary Information:**

The online version contains supplementary material available at 10.1186/s12883-025-04087-7.

## Background

Migraine is a primary headache, and it is one of about 200 recognized headache diseases [1]. The global estimate of migraine prevalence is 14 to 15%, and the migraine-attributed burden accounts for 4.9% of global population ill health quantified in years lived with disability [2]. Migraine is a major cause of disability and negatively impacts health-related quality of life (HRQoL) of patients [3, 4]. The effects of migraine on HRQoL are harmful and extensive, with adverse effects on work as well as physical, emotional, and social aspects of daily life [5, 6]. Several studies have linked higher headache frequency to worse health status [7–10]. Chronic migraine (CM) is a complex neurological disorder defined as ≥ 15 headache days per month, with ‘ ≥ 8 days.’ Fewer than 5% of those with CM traversed the three barriers to basic care: consulting an HCP, obtaining an accurate diagnosis, and receiving both preventive and acute treatments [11]. OnabotulinumtoxinA [12] is indicated for preventing headaches in adult patients with CM. In the pivotal phase 3 PREEMPT trials, treatment consisted of intramuscular injections of 155 U – 195 U of onabotulinumtoxinA distributed among 7 head and neck regions innervated by the trigeminal neurovascular system [13]. OnabotulinumtoxinA anti-nociceptive effects are thought to involve the decreased release of proinflammatory neurotransmitters and neuropeptides that transmit nociceptive pain such as substance P, calcitonin gene-related peptide, and glutamate from primary afferent fibers. In addition, onabotulinumtoxinA also inhibits the insertion of pain-sensitive ion channels such as transient receptor potential cation channel subfamily V member 1 (TRPV1) into synaptic membranes [14–27]. In the US, the labelled dose is 155 U for prophylaxis treatment of CM. However, in Canada and Europe, 155 U – 195 U is the licensed dose, and since its approval for CM in 2010 there have been numerous clinical and real-world studies reporting on the higher doses of 156 U – 195 U [13, 28–31].

The REal-life use of botulinum toxin for the symptomatic treatment of adults with chronic migraine, measuring healthcare resource utilization, and Patient-reported OutcomeS observed in practicE (REPOSE) study was a 24-month observational study conducted across multiple sites in Europe. REPOSE utilized patient- and physician-reported outcomes to evaluate the effectiveness and safety of real-life, long-term use of onabotulinumtoxinA for CM and assessed it’s utilization in routine clinical practice. The outcomes reported by both patients and physicians in the REPOSE study showed that onabotulinumtoxinA treatment for CM led to a sustained decrease in headache-day frequency, significant improvement in quality of life measures, and a reduction in healthcare resource utilization with onabotulinumtoxinA treatment for CM [13, 28–30]. The objective of this REPOSE study analysis was to characterize real-world dose utilization of onabotulinumtoxinA and to evaluate patient outcomes based on the onabotulinumtoxinA doses administered – 155 U or the 156 U – 195 U dosing range.

## Methods

### Study design

The design of the REPOSE study (NCT01686581) has been previously detailed in a publication [32]. In summary, REPOSE was a 24-month, observational, prospective, open-label study involving patients prescribed onabotulinumtoxinA for the treatment of CM. Ethics approval was obtained by all study investigators from their respective ethics committees before the study began. REPOSE was conducted in compliance with the International Conference on Harmonization Guideline for Good Clinical Practice.

Eligible patients were adult men and women aged 18 years or older who were prescribed onabotulinumtoxinA for the symptomatic treatment of CM. Patients were excluded if they had received any botulinum toxin serotype within 26 weeks prior to enrollment, were participating in Allergan's Botox CM Post-Authorization Safety Study (PASS) or had contraindications for onabotulinumtoxinA treatment.

Investigators were asked to refer to the Summary of Product Characteristics (SmPC) for details on contraindications (Sect. 4.3), warnings (Sect. 4.4), and pregnancy and lactation (Sect. 4.6). To best capture real-world clinical practice, no additional specific exclusion criteria were applied in this study. Patients were not excluded for having received acute or other preventive treatments prior to study enrollment and were allowed to continue these treatments, as needed, during the study. All patients gave written informed consent before enrollment.

### OnabotulinumtoxinA treatment

Treating physicians were instructed based on the injection paradigm outlined in the SPC and the PREEMPT study protocol, which includes administering 155 U of onabotulinumtoxinA across 31 injection sites every 12 weeks, with the option based on the treating physicians discretion to administer an additional 40 U over 8 injection sites following the follow-the-pain strategy, for a maximum total dose of 195 U. However, adherence to this paradigm was not mandatory. During each visit, the total dose per treatment session, along with the total number and specific locations of injection sites, was documented for all patients. The most common deviation noted was in the treatment interval. A majority of patients (79.1%) received treatment at intervals longer than 13 weeks, and nearly half (46.0%) received treatment at intervals exceeding 16 weeks at least once. We did not record reasons for deviations in the treatment interval, as asking about this might have unintentionally influenced treatment practices and led to more physicians adhering to the recommended protocol.

### Study outcomes

Patient demographics, medical and headache history were recorded at the baseline visit. Patient-reported headache day frequency, the Migraine Specific Quality of Life Questionnaire (MSQ) v2.1 [33], the EuroQol 5-Dimension Questionnaire (EQ-5D) [34], EQ-5D total score and health state were documented at baseline and each onabotulinumtoxinA administration visit. Physician and patient assessments at each follow-up visit included satisfaction with treatment (insufficient, moderate, good, very good) and tolerability of treatment (poor, moderate, good, very good).

Adverse drug reactions (ADRs) and serious ADRs were documented throughout the study duration in the electronic case report form. An ADR was defined as a noxious and unintended response to any treatment administered at a therapeutic dose where a causal relationship between a treatment and an adverse event was at least a reasonable possibility. Serious ADRs were any adverse drug reaction occurring at any dose that resulted in death, a life-threatening adverse event, inpatient hospitalization or prolongation of existing hospitalization, a persistent or significant disability/incapacity, or a congenital anomaly/birth defect.

### Statistical analysis

The population used for analysis for demographic, effectiveness, and safety data included participants from the safety analysis set (participants that received at least 1 dose of onabotulinumtoxinA) stratified into two groups by treatment dose at ≥ 4 visits: 155 U onabotulinumtoxinA and 156 U − 195 U onabotulinumtoxinA. Patients received onabotulinumtoxinA treatment approximately every 12 weeks, as determined by their physicians’ discretion, following the guidelines in the SmPC and the PREEMPT injection paradigm within the SmPC [35]. Eight participants met the inclusion criteria for both dosage groups and were excluded from the analysis. In these patients, dosages changed throughout the 24-month period where they each were treated with at least 4 cycles of 155 U and at least 4 cycles of 156 U – 195 U. Administration visits were defined as visits during which onabotulinumtoxinA was injected.

Change from baseline in the effectiveness variables were evaluated using a paired t-test. Descriptive statistics are shown for continuous variables, while percentages are reported for categorical data.

If the answer to an MSQ questionnaire question was missing, the affected dimension score was set to missing, and the total score was set to missing.

## Results

### Baseline characteristics

A total of 641 participants were enrolled at 77 centers in 7 European countries: Germany, UK, Italy, Norway, Spain, Russia, and Sweden. Of those 218 participants received 155 U at 4 or more treatment visits and 77 participants received 156 U – 195 U onabotulinumtoxinA at 4 or more treatment visits (of these, 24 received 195 U at 4 or more treatment visits).

Eight participants met the inclusion criteria for both 155 U and 156 U – 195 U dosage groups and were excluded from this analysis. Demographics and baseline clinical characteristics were similar between groups and are presented in Table 1.

**Table 1:** Demographics and baseline characteristics

	**155 U onabotulinumtoxinA**^**a**^	**156 U – 195 U onabotulinumtoxinA**^**a**^
	**(*n* = 218)**	**(*n* = 77)**
**Age, years (SD)**	45.1(11.9)	45.5 (11.3)
**Sex, n (%)**		
* Female*	188 (86.2)	68 (88.3)
* Male*	30 (13.8)	9 (11.7)
**Headache Days/Month, n (SD)**	21.5 (5.5)	21.5 (5.6)
**MSQ domain Score**		
* Role Function-Restrictive*	34.3 (17.6)^b^	35.3 (16.3)^c^
* Role Function-Preventive*	47.0 (22.3)^d^	50.4 (18.8)^c^
* Emotional Function*	37.9 (24.9)^b^	41.2 (24.2)^e^
**BMI mean (SD)**	25.08 (5.464)	24.19 (3.796)
**Country n (%)**		
* Germany*	94 (43.1)	49 (63.6)
* Italy*	1 (0.5)	15 (19.5)
* Norway/Sweden*	4 (1.8)	3 (3.9)
* Russia*	8 (3.7)	9 (11.7)
* Spain*	52 (23.9)	1 (1.3)
* UK*	59 (27.1)	0
**Ongoing Psychiatric comorbidities n (%)**		
* Anxiety*	17 (7.8)	4 (5.2)
* Depression*	27 (12.4)	7 (9.1)
* Bipolar disorder*	1 (0.5)	1 (1.3)

^a^Participants treated with onabotulinumtoxinA in at least 4 visits; ^b^*n* = 213; ^c^*n* = 75; ^d^*n* = 209; ^e^*n* = 76

Data are n (SD) or n (%). MSQ, Migraine-Specific Quality of Life Questionnaire; SD, standard deviation; U, units.

## Effectiveness

**Fig. 1:**
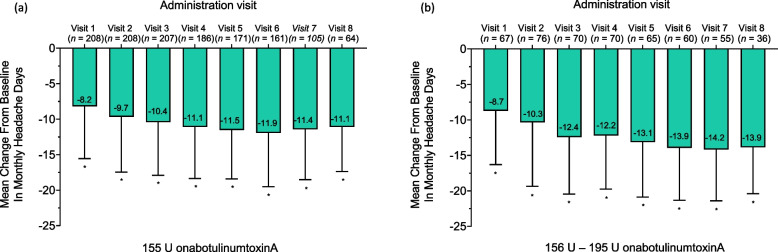
Mean (SD) change from baseline in frequency of headache days. Mean (SD) change from baseline in frequency of headache days. Patient-reported estimated number of days per month with a headache (≥ 4 h) at each administration visit 1 through 8. Patients treated with (**a**) 155 U onabotulinumtoxinA or (**b**) 156 U – 195 U at 4 or more visits. * *p* < 0.001 paired t- test for change vs baseline. h, hours; SD, standard deviation; U, units

At baseline, the mean (SD) headache day frequency in the 155 U group was 21.5 (5.5) days (Fig. 1(a)). Treatment with 155 U onabotulinumtoxinA resulted in a mean reduction of 8.2 headache days at administration visit 1 and 11.1 headache days at administration visit 8. At baseline, the mean (SD) headache day frequency in the 156 – 195 U group was 21.5 (5.6) (Fig. 1(b)). Treatment with 156 U – 195 U onabotulinumtoxinA resulted in a mean reduction of 8.7 to 14.2 headache days per month across all time points.

**Fig. 2:**
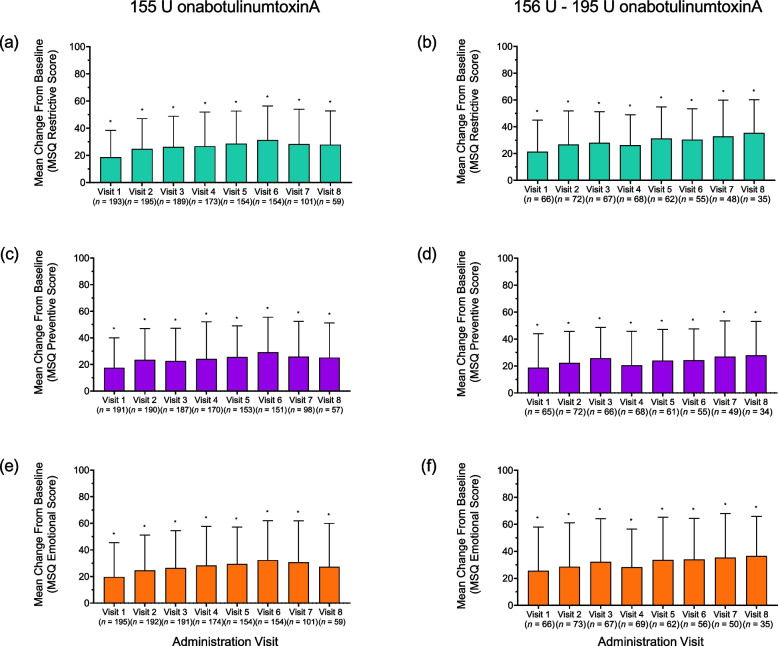
Mean (SD) change from baseline in MSQ dimensions. Mean (SD) change from baseline in MSQ dimensions at administration visit 1 through 8. (**a**-**b**) Role-function restrictive score; (**c**-**d**) Role-function preventive; (**e**–**f**) Emotional function score. Patients treated with 155 U onabotulinumtoxinA (left) or 156–195 U (right) at 4 or more visits. MSQ = Migraine Specific Quality of Life Questionnaire; SD, standard deviation. * *p* < 0.001 paired t- test for change vs baseline

Treatment with 155 U and 156 U – 195 U onabotulinumtoxinA resulted in increased MSQ scores across administration visits in all role function domains compared with baseline (Fig. 2).

**Fig. 3:**
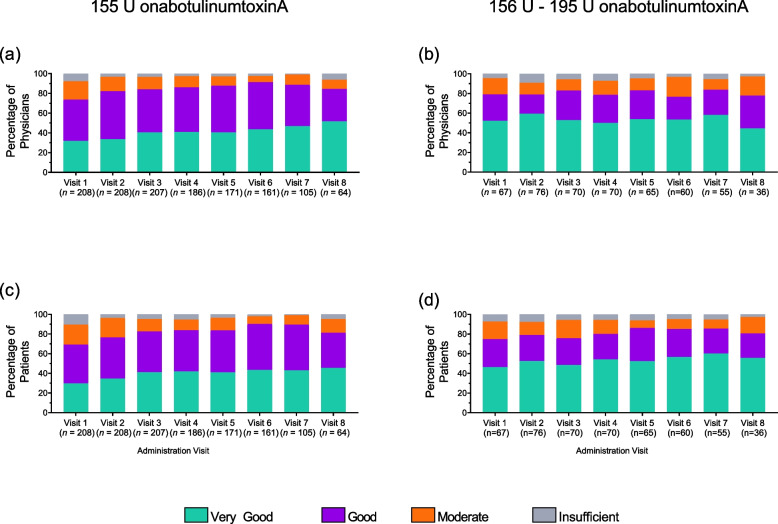
Satisfaction with onabotulinumtoxinA treatment. Physician (**a**-**b**) and patient (**c**-**d**) satisfaction (very good, good, moderate, or insufficient) at administration visit 1 through 8. Patients treated with 155 U onabotulinumtoxinA (left) or 156 U –195 U (right) at 4 or more visits. U, units

Satisfaction with onabotulinumtoxinA treatment was assessed by patients and physicians at each follow-up visit and was high throughout the study period. The frequency of the treatment satisfaction levels (insufficient, moderate, good, very good) are reported (Fig. 3).

**Fig. 4:**
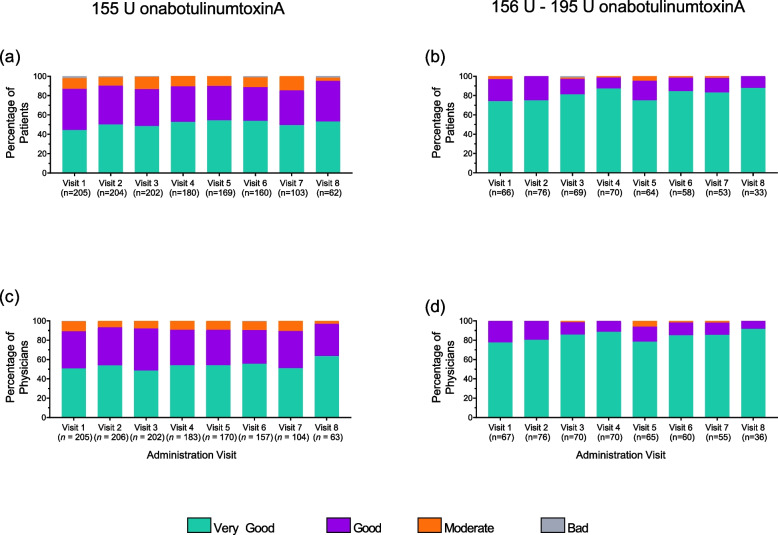
Evaluation of onabotulinumtoxinA treatment tolerability. Evaluation of onabotulinumtoxinA treatment tolerability. Patient (**a**-**b**) Physician (**c**-**d**) evaluation of tolerability (very good, good, moderate, or bad) at administration visit 1 through visit 8. Patients treated with 155 U onabotulinumtoxinA (left) or 156 – 195 U (right) at 4 or more visits. U, units

Over 80% of patients treated with onabotulinumtoxinA 155 U and 156 U – 195 U rated treatment satisfaction as ‘very good’ or ‘good’ at administration visit 8. The majority of patients rated tolerability as ‘very good’ or ‘good’ at administration visit 8 (155 U 95.2%; 156 U – 195 U 100%) (Fig. 4). Similar to patients, physicians reported high rates for satisfaction (155 U 84.4%; 156 U – 195 U 77.8%) (Fig. 3) and tolerability (155 U 96.8%; 156 U – 195 U 100%) (Fig. 4).

Results of the EuroQol 5-Dimension Questionnaire (EQ-5D) demonstrated that both dose groups exhibited an improvement trend from baseline in all EQ-5D dimensions, as indicated by the proportions of patients per level of perceived problems at administration visit 8 (Supplementary Fig. 1). Improvement was observed in both the EQ-5D total score and health state score at administration visit 8. The mean (SD) change from baseline in EQ-5D total score was 0.24 (0.39) in the 155 U group and 0.31 (0.40) in the 156 U – 195 U group from mean baseline scores of 0.49 (0.38) and 0.40 (0.36), respectively. For the health state score, the mean (SD) change from baseline was 22.84 (30.9) in the 155 U group and 22.0 (21.02) in the 156 U – 195 U from mean baseline scores of 46.9 (27.1) and 47.6 (20.1), respectively (Supplementary Fig. 2).

## Safety

Adverse drug reactions were reported in 51 of 218 (23.4%) patients in the 155 U group and 10 of 77 (13.0%) in the 156 U – 195 U group (Table 2). Serious ADRs occurred in 3 (1.4%) and 1 (1.3%) patient in the 155 U and 156 U – 195 U groups, respectively (Table 2). The most frequently reported ADR across both dose groups were eyelid ptosis (5.4%), followed by neck pain (2.8%), and musculoskeletal stiffness (2.7%). These safety results are consistent with ADRs reported in phase 3 clinical trials (PREEMPT) [13].

**Table 2:** Summary of adverse drug reactions occurring in the safety population

	**155 U onabotulinumtoxinA**	**156–195 U onabotulinumtoxinA**
	**(*n* = 218)**	**(*n* = 77)**
**Adverse drug reaction**	51 (23.4)	10 (13.0)
*None*	167 (76.6)	67 (87.0)
*Mild*	24 (11.0)	5 (6.5)
*Moderate*	18 (8.3)	3 (3.9)
*Severe*	9 (4.1)	2 (2.6)
**Serious adverse drug reaction**	3 (1.4)	1 (1.3)

^a^Participants treated with onabotulinumtoxinA in at least 4 Visits.

Data are n (%). U, units.

## Discussion

Migraine is the second leading cause of disability according to the Global Burden of Disease Study [36]. Patients with chronic migraine (CM) experience disabling migraine attacks that significantly affect their quality of life and interfere with their ability to perform daily activities [37]. CM is also associated with a greater frequency and severity of migraine-associated disability [38].

The objective of the REPOSE study was to provide observational data regarding the effectiveness, safety, tolerability, and utilization of onabotulinumtoxinA for the preventive treatment of CM over a 2-year period, measuring healthcare resource utilization and patient-reported outcomes observed in clinical practice. OnabotulinumtoxinA received FDA approval in October 2010, as an injectable for the preventive treatment of CM, with a recommended dose of 155 units. Despite the approval of 155 U, in both pivotal phase 3 PREEMPT studies, health care providers were given the discretion to inject up to 40 additional units of onabotulinumtoxinA (up to 195 U) into 8 additional sites (up to 39 sites total) to maximize treatment benefits. The real-world findings from the REPOSE study comparing two treatment doses 155 U vs 195 U, demonstrate that 156 U – 195 U onabotulinumtoxinA is efficacious and safe, consistent with findings from the PREEMPT clinical trials. Phase 3 PREEMPT trials provided evidence of the safety and efficacy of 155 U – 195 U of onabotulinumtoxinA for the preventive treatment of CM in adults [39, 40]. In the REPOSE study, Patients treated with doses of 156 U – 195 U had a statistically significant decrease in headache day frequency as compared to baseline at all time points evaluated in the study (visit 1 through visit 8).

Across the 10 follow-up time points, treatment with 156 U – 195 U onabotulinumtoxinA continued to show efficacy, resulting in a change of 8.9 to 17.2 fewer headache days/month. In comparison, those treated with 155 U reported a decrease ranging from 8.2 to 13.6 days/month. These differences were clinically meaningful. Visits 9 and 10 had very few patients, therefore, these 2 visits were not included in the analysis.

In the REPOSE study, 36% of patients received doses greater than 155 U, highlighting the real-world use of doses > 155 U outside United States and further underscoring the potential need for higher doses for the treatment of CM for some patients. Interestingly, it has been reported that over two-thirds of clinicians in the United States might be altering the PREEMPT protocol to ‘follow the pain’, based on the survey results [41]. Additional research is needed to validate those findings and capture self-reported satisfaction with onabotulinumtoxinA treatments across the 155 U – 195 U dosing range in the United States.

In addition to REPOSE, several other studies have explored the effectiveness of onabotulinumtoxinA doses higher than 155 U for the treatment of CM. The PREDICT study, a Canadian, multicenter, prospective, observational standard of care study, demonstrated that adults with CM who received 155 U – 195 U of onabotulinumtoxinA over a 2-year period experienced a significant improvement in headache days per month and improvement in MSQ scores compared to baseline [31]. A 2-year open-label prospective study also showed superior efficacy of 195 U of onabotulinumtoxinA compared to 155 U in chronic migraine patients with medication overuse headache (MOH) during a 2-year treatment period [42]. Similarly, a retrospective paired comparison study found that increasing the dose from 150 to 200 U over three rounds of injections led to a significant reduction in headache days and severe headache days in patients with CM [43]. Two additional retrospective studies have suggested that increasing the units of onabotulinumtoxinA, further supporting that increasing the dose of onabotulinumtoxinA may also increase the duration of effect [44, 45]. These findings collectively suggest that higher doses (> 155 U) of onabotA are safe and efficacious, similarly to 155 U dose, and could be a useful tool in the HCP armamentarium when optimizing individual treatment outcomes of chronic migraine patients.

### Limitations/Generalizability

Participant-reported outcomes such as headache-day frequency, MSQ, and EQ-5D were self-reported and are based on participant recollection. Poor recollection may result in incomplete and/or missing data. Nevertheless, outcome data supported significant improvement in quality of life measures, similar to previous clinical and real-life studies [46–48].

Discontinuation of onabotulinumtoxinA treatment in REPOSE due to lack of efficacy may have led to an enriched patient population, potentially confounding the results, particularly regarding patient outcomes over time. Observational studies conducted in real-life clinical settings come with inherent limitations, as they typically involve less stringent monitoring and rely heavily on healthcare professionals to accurately record study-related data. Additionally, these studies reflect real-world treatment conditions, where many patients are likely using concomitant preventive medications. These aspects should be considered when interpreting the discontinuation data.

A notable limitation of the study is the lack of understanding whether higher doses of OnabotulinumtoxinA translate into better efficacy. The PREEMPT trial was not specifically designed to investigate this aspect. Furthermore, there is a lack of clarity regarding which patients would be the most suitable candidates for higher than 155 U doses, and which specific 'follow the pain' injection paradigm would be most suitable to improve individual outcomes.

Real-world studies due to their observational nature are subject to limitations, such as recall bias, lack of formal protocol requirements and exclusion criteria. To understand real-world treatment practices, this observational study did not use any formal protocol requirements or exclusion criteria. Additionally, the study was restricted to 7 countries (Germany, UK, Italy, Norway, Spain, Russia, and Sweden), with most patients from Germany, and the generalizability of data to other countries/patient populations should be taken with caution.

A real-life observational study offers outcomes that enhance the understanding of treatment use in clinical practice. In the REPOSE Study, treating physicians were trained according to the injection paradigm described in the PREEMPT study protocol and recommended in the SmPC. Treatments were not mandated but were provided at the participating physicians' discretion according to their clinical judgment and local standards of medical care.

## Conclusions

Real-world findings from the open-label, prospective, noninterventional REPOSE study support effectiveness, safety, and tolerability of both the 155 U and 195 U doses of onabotulinumtoxinA as a treatment option for CM patients, consistent with the PREEMPT clinical trials. In the REPOSE study, treatment with onabotulinumtoxinA for both the 155 U and the 155 U – 195 U doses demonstrated sustained reduction in frequency of headache days and significant improvement in the quality of life measures (MSQ and EQ-5D) across 8 treatment cycles. Self-reported satisfaction with the treatment across both doses was rated as “good” or “very good” by the majority (> 75%) of patients and physicians. Treatment across all onabotulinumtoxinA doses (155 U – 195 U) tested in the REPOSE study was safe and generally well-tolerated, with no new safety signals identified. In the REPOSE study, the dose and number of onabotulinumtoxinA injection sites used were consistent with licensed recommendations. Most sessions involved doses ranging from 155 to 195 U, with a median of 31 injection sites across all follow-up visits. This supports the consideration for higher doses, up to 195 units, of chronic migraine (CM) patients as part of clinical practice for migraine management.

## Supplementary Information

Supplementary Material 1.

Supplementary Material 2.

## Data Availability

AbbVie is committed to responsible data sharing regarding the clinical trials we sponsor. This includes access to anonymized, individual, and trial-level data (analysis data sets), as well as other information (e.g., protocols, clinical study reports, or analysis plans), as long as the trials are not part of an ongoing or planned regulatory submission. This includes requests for clinical trial data for unlicensed products and indications. These clinical trial data can be requested by any qualified researchers who engage in rigorous, independent, scientific research, and will be provided following review and approval of a research proposal, Statistical Analysis Plan (SAP), and execution of a Data Sharing Agreement (DSA). Data requests can be submitted at any time after approval in the US and Europe and after acceptance of this manuscript for publication. The data will be accessible for 12 months, with possible extensions considered. For more information on the process or to submit a request, visit the following link: https://vivli.org/ourmember/abbvie/ then select “Home”.

## Abbreviations

ADR: Adverse Drug Reaction
BOTOX®: OnabotulinumtoxinA
CM: Chronic Migraine
DSA: Data sharing agreement
EQ-5D: EuroQol 5-Dimension Questionnaire
FDA: Food and Drug Administration
HCP: Health Care Provider
HRQoL: Health-Related Quality of Life
HIS: International Headache Society
MOH: Medication Overuse Headache
MSQ: Migraine Specific Quality of Life Questionnaire
PASS: Post-Authorization Safety Study
PREEMPT: Phase 3 Research Evaluating Migraine Prophylaxis Therapy
SAP: Statistical Analysis Plan
SPC: Summary of Product Characteristics
