# Supplementary material for: Real-World Safety and Efficacy of 156 U – 195 U OnabotulinumtoxinA in Adults With Chronic Migraine: Results From the REPOSE Study
Source: BMC Neurol. 2025 May 6;25:197. doi: 10.1186/s12883-025-04087-7 (PMC12053858; doi:10.1186/s12883-025-04087-7)
Supplement: Supplementary file 1 — Supplementary Material 1. [file 12883_2025_4087_MOESM1_ESM.pptx]

## Slide 1
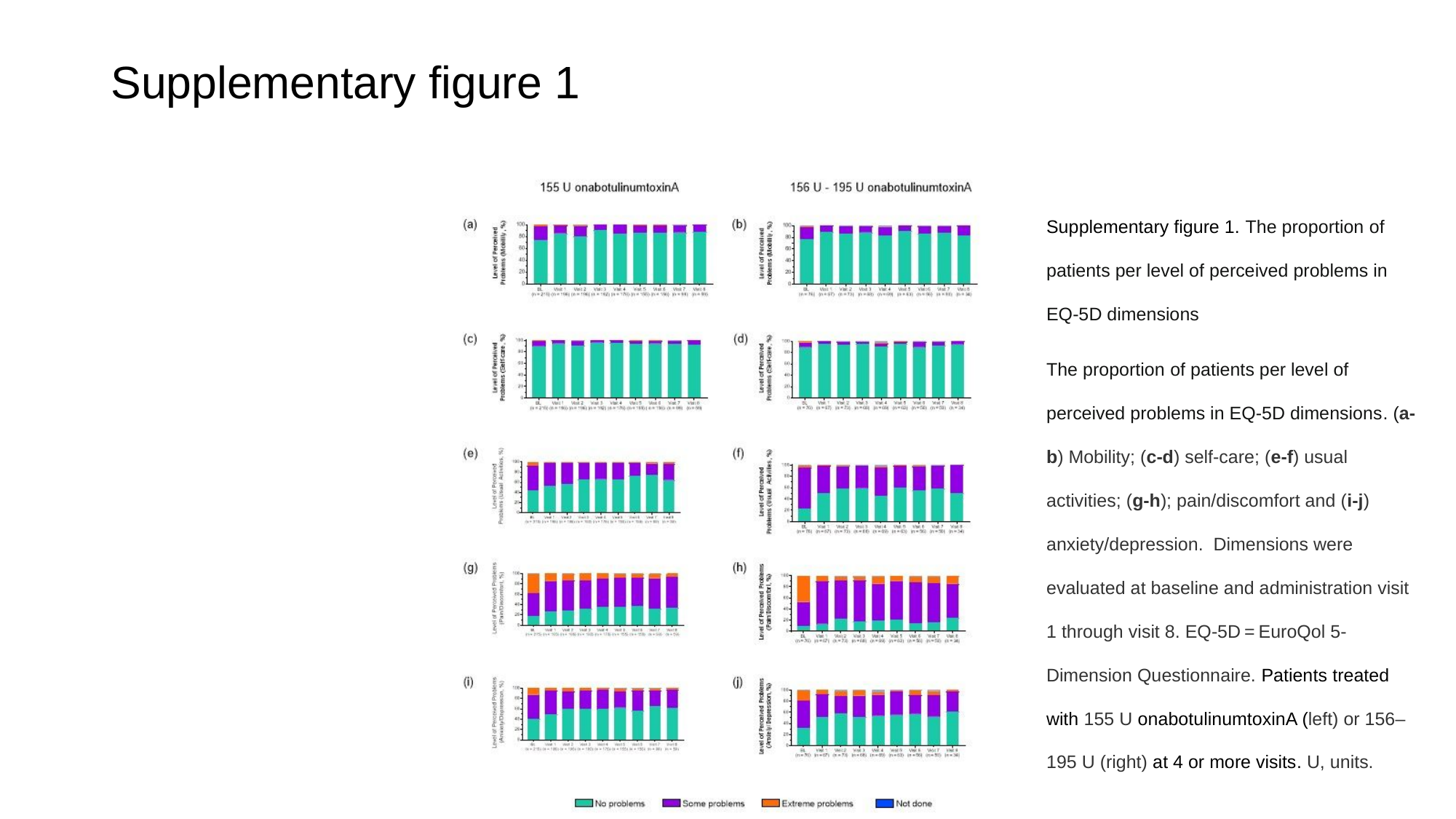

# Supplementary figure 1
Supplementary figure 1. The proportion of patients per level of perceived problems in EQ-5D dimensions
The proportion of patients per level of perceived problems in EQ-5D dimensions. (a-b) Mobility; (c-d) self-care; (e-f) usual activities; (g-h); pain/discomfort and (i-j) anxiety/depression.  Dimensions were evaluated at baseline and administration visit 1 through visit 8. EQ-5D = EuroQol 5-Dimension Questionnaire. Patients treated with 155 U onabotulinumtoxinA (left) or 156–195 U (right) at 4 or more visits. U, units.
