# Supplementary material for: Real-World Safety and Efficacy of 156 U – 195 U OnabotulinumtoxinA in Adults With Chronic Migraine: Results From the REPOSE Study
Source: BMC Neurol. 2025 May 6;25:197. doi: 10.1186/s12883-025-04087-7 (PMC12053858; doi:10.1186/s12883-025-04087-7)
Supplement: Supplementary file 2 — Supplementary Material 2. [file 12883_2025_4087_MOESM2_ESM.pptx]

## Slide 1
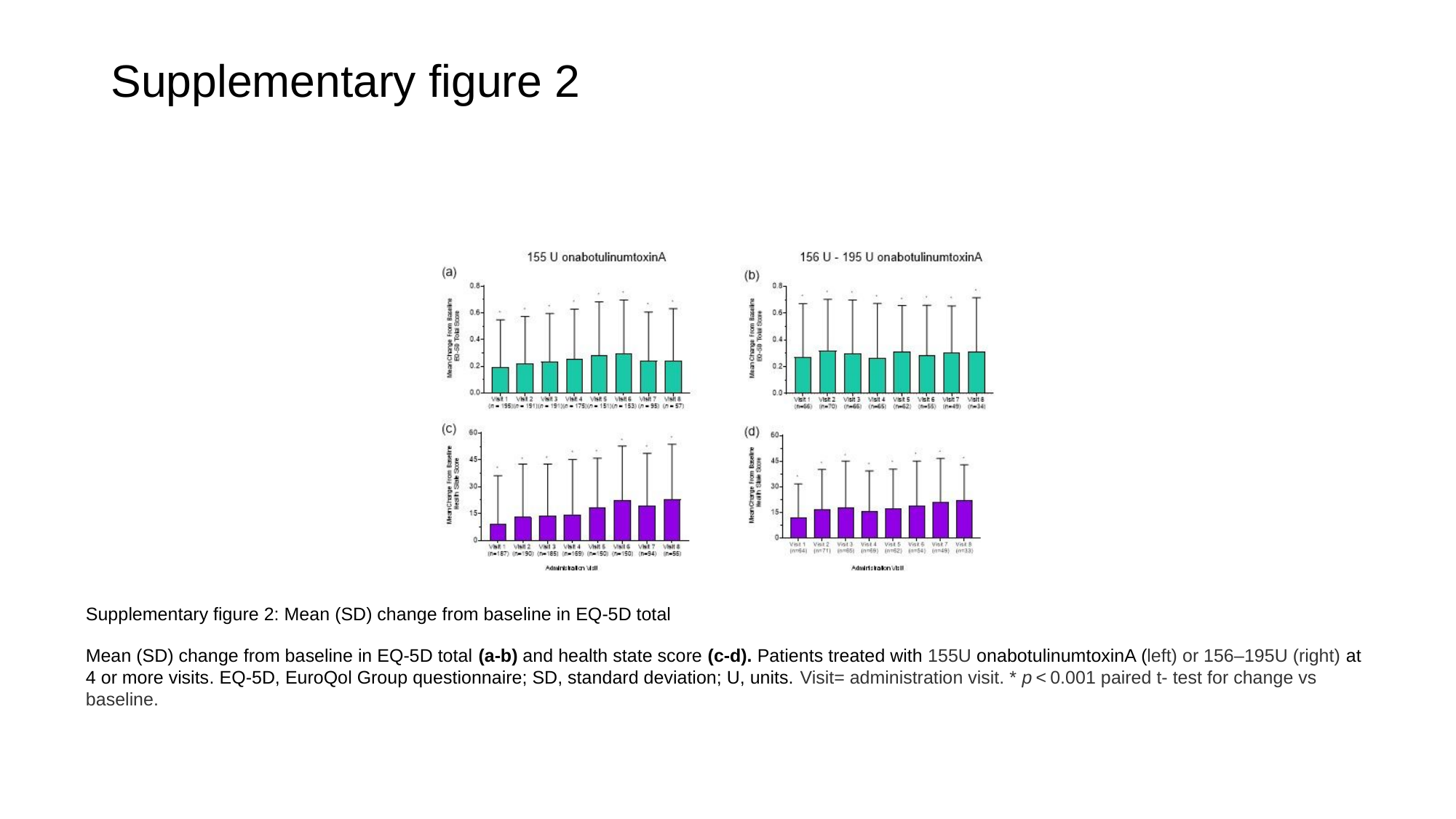

# Supplementary figure 2
Supplementary figure 2: Mean (SD) change from baseline in EQ-5D total
Mean (SD) change from baseline in EQ-5D total (a-b) and health state score (c-d). Patients treated with 155U onabotulinumtoxinA (left) or 156–195U (right) at 4 or more visits. EQ-5D, EuroQol Group questionnaire; SD, standard deviation; U, units. Visit= administration visit. * p < 0.001 paired t- test for change vs baseline.
